# Polyhydroxy structure orchestrates the intrinsic antibacterial property of acrylamide hydrogel as a versatile wound-healing dressing

**DOI:** 10.3389/fbioe.2024.1396892

**Published:** 2024-04-24

**Authors:** Lu Zhang, Yu-Jiao Tang, Wen-Qing Zhang, Jian Wang, Yu-Jian Cai, Tian-Yi Qin, Deteng Zhang, Zhao-Hui Wang, Ya-Long Wang

**Affiliations:** ^1^ State Key Laboratory of Digital Medical Engineering, Key Laboratory of Biomedical Engineering of Hainan Province, School of Biomedical Engineering, Hainan University, Sanya, Hainan, China; ^2^ Institute of Neuroregeneration and Neurorehabilitation, Qingdao University, Qingdao, China

**Keywords:** antibacterial hydrogels, wound healing, inherent antimicrobial, polyhydroxy, acrylamide

## Abstract

Hydrogel is considered as a promising candidate for wound dressing due to its tissue-like flexibility, good mechanical properties and biocompatibility. However, traditional hydrogel dressings often fail to fulfill satisfied mechanical, antibacterial, and biocompatibility properties simultaneously, due to the insufficient intrinsic bactericidal efficacy and the addition of external antimicrobial agents. In this paper, hydroxyl-contained acrylamide monomers, N-Methylolacrylamide (NMA) and N-[Tris (hydroxymethyl)methyl] acrylamide (THMA), are employed to prepare a series of polyacrylamide hydrogel dressings xNMA-yTHMA, where x and y represent the mass fractions of NMA and THMA in the hydrogels. We have elucidated that the abundance of hydroxyl groups determines the antibacterial effect of the hydrogels. Particularly, hydrogel 35NMA-5THMA exhibits excellent mechanical properties, with high tensile strength of 259 kPa and large tensile strain of 1737%. Furthermore, the hydrogel dressing 35NMA-5THMA demonstrates remarkable inherent antibacterial without exogenous antimicrobial agents owing to the existence of abundant hydroxyl groups. Besides, hydrogel dressing 35NMA-5THMA possesses excellent biocompatibility, in view of marginal cytotoxicity, low hemolysis ratio, and negligible inflammatory response and organ toxicity to mice during treatment. Encouragingly, hydrogel 35NMA-5THMA drastically promote the healing of bacteria-infected wound in mice. This study has revealed the importance of polyhydroxyl in the antibacterial efficiency of hydrogels and provided a simplified strategy to design wound healing dressings with translational potential.

## 1 Introduction

The skin is the main defense mechanism of the human body, which is the protective barrier between the external environment and the internal organs. It protects various tissues and organs from physical, mechanical, chemical and pathogenic microbiological attacks (Wang et al., 2021; Ding et al., 2023; Qiao et al., 2023). At the same time, the skin is also involved in important physiological activities and plays a key role in maintaining homeostasis in the body. But it is vulnerable to external injuries, such as severe friction and sharp cuts, which may lead to skin trauma and loss of barrier function and be attacked by bacteria, fungi, viruses and other microorganisms in the external environment (Shortridge and Flamm, 2019; Zhao et al., 2020). Although mild skin injuries can heal naturally, the healing process of severe skin injuries may be disturbed by many factors, such as bacterial infection, chronic inflammation, diabetes, etc., which make it difficult to heal. In some cases, infection may further worsen and even lead to systemic infections, such as septicemia (Zhou et al., 2021; Song et al., 2024). Therefore, it is highly desirable to develop effective skin wound dressing to prevent infection.

Traditional wound dressings, such as gauze, cotton, bandages, etc., have been widespreadly used in clinical treatment (Wei et al., 2021; Priya et al., 2024). However, these traditional dressings lack antibacterial properties and need to be changed frequently, easily destroying new skin tissue due to easy adhere to tissue and increasing the risk of infection (Zhou et al., 2022; Zhou et al., 2023a). In recent years, new wound dressings with excellent antibacterial properties, good biocompatibility, and promotion of wound healing have been gradually developed (Zhang et al., 2021). Among them, hydrogel materials have gradually become a research hotspot in wound dressings due to their unique water-rich three-dimensional network structure, mechanical properties similar to soft tissue, easy drug-loading structure, and rich functional groups (Farahani and Shafiee, 2021; Guo et al., 2021). Commonly hydrogel used for wound dressings include natural polysaccharides (chitosan, sodium alginate, and so on) and polyacrylamides et al. (Peng et al., 2022). Some chemical cross-linking agents, such as glutaraldehyde, etc., show obvious cytotoxicity (Guo et al., 2021; Giulia Mugnaini, 2023). Hence, physically cross-linking hydrogels with stable three-dimensional network structures, which formed based on supramolecular interactions (hydrogen bonds, ionic bonds, complexation, etc.), are ideal candidates for developing hydrogel antimicrobial dressings. According to whether the hydrogel is loaded with exogenous antimicrobial agents, hydrogel antibacterial dressings can be divided into hydrogel wound dressings loaded with antibacterial agents and inherent antibacterial hydrogel wound dressings (Li et al., 2023; Zhao et al., 2023). Wound dressings loaded with antimicrobials need to add exogenous antimicrobial agents (antibiotics, metal ions, cationic antimicrobials, natural antimicrobials, etc.) to the hydrogel substrate. In general, the composition of these hydrogel antibacterial dressing is complex (Hu et al., 2018; Zhang et al., 2023b; Zhang et al., 2024). Moreover, the drug resistance, safety, addition amount and release rate of antimicrobial agents need to be considered. It is worth noting that the addition of exogenous antimicrobial agents usually affects the mechanical properties of the hydrogel substrate (Supplementary Tabel S1) (Li et al., 2018; Zhou et al., 2023b; Moradi et al., 2023). The inherent antibacterial hydrogel wound dressings is prepared by hydrogel materials with antibacterial activity. However, at present, most of the inherent antibacterial hydrogel wound dressings also introduce cationic groups into the hydrogel molecular chain or introduce metal ions through ion coordination. However, excessive cations and metal ions usually cause irritation and damage to normal cells, causing allergies and other symptoms (Cui et al., 2023; Li et al., 2024). In view of the fact that it is difficult for existing hydrogel dressings to achieve a satisfactory balance between multiple functions. Therefore, it is urgent to develop new type of physically cross-linking inherent antibacterial hydrogel wound dressings with simple components, non-cationic type, excellent mechanical properties and high biocompatibility.

Commonly natural polysaccharide hydrogels containing many hydroxyl groups in the molecular chain, such as chitosan, sodium alginate, possess certain antibacterial properties (Zhang et al., 2023a; El-Sayed et al., 2023; Lin et al., 2023). However, Due to its poor solubility in water, the prepared hydrogels possess low hydroxyl content and no abundant hydrogen bonding interactions, resulting in weak mechanical properties and unsatisfactory antibacterial activity (Dong et al., 2022; Jiang et al., 2022). Polyacrylamide hydrogel containing a large number of amide groups is prepared by polymerization with high concentration of acrylamide monomer. Polyacrylamide hydrogel exhibit good mechanical properties due to abundant hydrogen bonding interactions deriving from amide groups (Zhao et al., 2021; Liu et al., 2023; Wang et al., 2023). Unfortunately, its antibacterial performance is weak. The integration of hydroxyl groups into polyacrylamide hydrogel is expected to enhance its antibacterial properties and mechanical properties. In this paper, acrylamide hydrogel monomers N-Methylolacrylamide (NMA) and N-[Tris (hydroxymethyl)methyl] acrylamide (THMA), containing hydroxyl groups were selected to prepare non-ionic, physically cross-linking, hydroxyl-rich hydrogel dressings xNMA-yTHMA with different monomer content by free radical polymerization. The micro-morphology, mechanical properties, swelling, biocompatibility and antibacterial activity of hydrogels were verified by experiments. Hydroxyl-rich xNMA-yTHMA hydrogels not only show excellent mechanical properties and antibacterial properties, but also possess high biocompatibility. This provides a new strategy for development of hydrogel dressings with high biocompatibility, excellent mechanical properties and strong antibacterial properties.

## 2 Results and discussion

### 2.1 Preparation and mechanical properties of hydrogels

**SCHEME 1:**
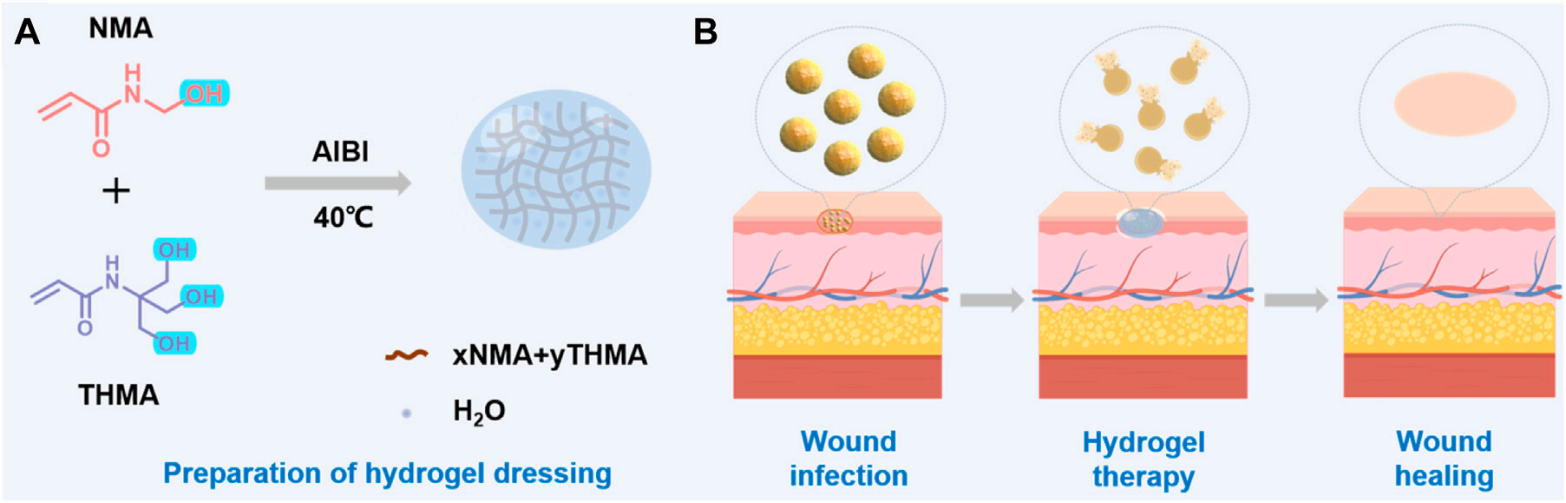
**(A)** Diagram of the synthesis of hydrogel. **(B)** Schematic diagram of skin wound healing.

A schematic diagram illustrating the process of preparing the composite hydrogel by N-Methylolacrylamide (NMA) and N-[Tris (hydroxymethyl)methyl] acrylamide (THMA), was presented in Scheme 1. NMA and THMA were dissolved in PBS and then the initiator 1,2-Bis(2-(4,5-dihydro-1H-imidazole-2-yl)propan-2-yl)diazene dihydrochloride (AIBI) was added to form a pre-polymer solution. The pre-polymer solution was added to a suitable mold and polymerized by heating at 40°C. According to the different concentrations of NMA and THMA monomers, different hydrogels were prepared and named as xNMA-yTHMA, where x and y represent the mass fraction of NMA and THMA in hydrogels, respectively. The shape and size of the mold can be designed according to the requirement. The detailed preparation procedure is displayed in the Supplementary Material.

**FIGURE 1:**
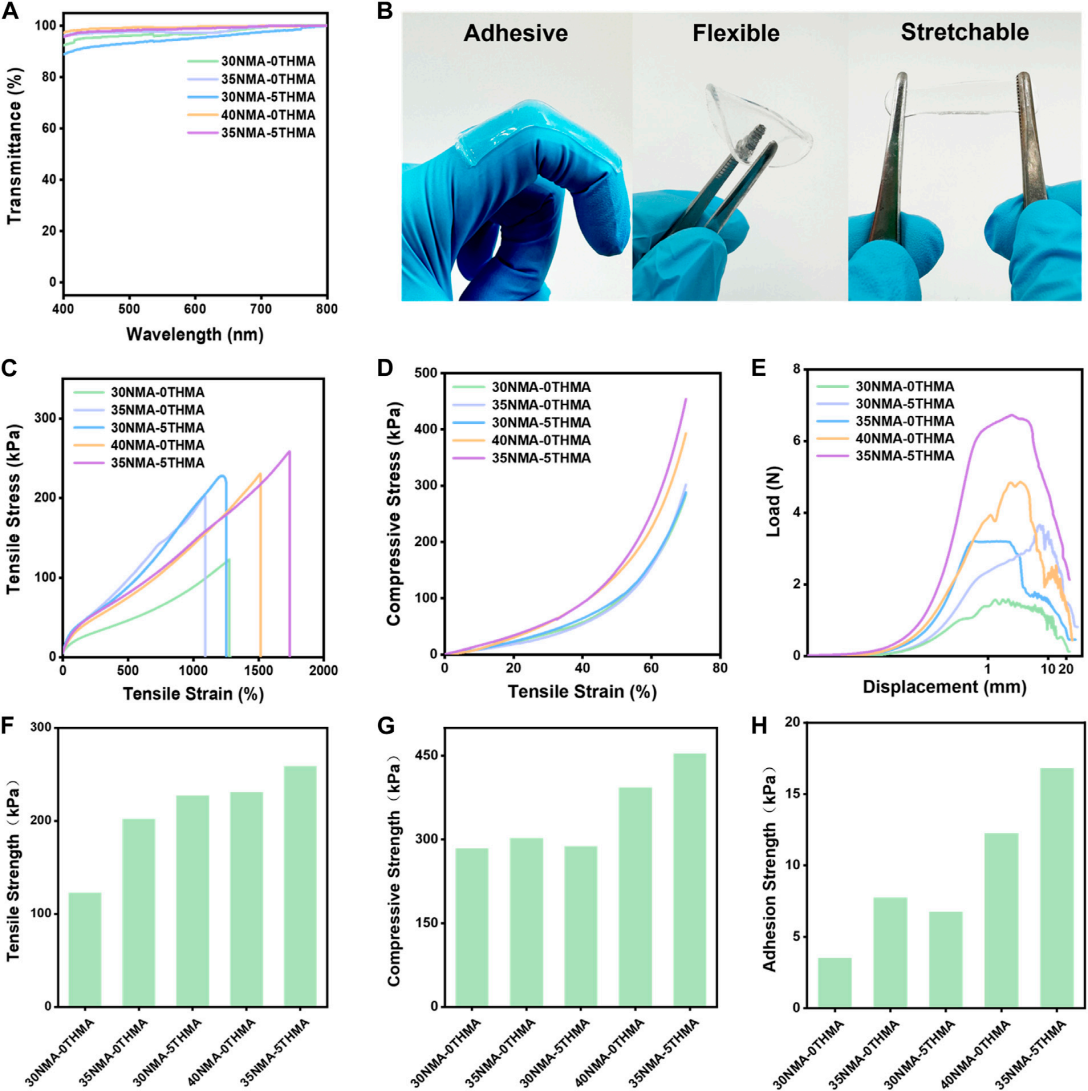
Mechanical properties of hydrogels. **(A)** The transmittance of different hydrogels within the visible wavelength ranging from 400 nm to 800 nm. **(B)** Images of hydrogel samples in adhesive, flexible and stretchable forms. **(C)** Tensile stress-strain curves of different hydrogels. **(D)** Compressive stress-strain curves of different hydrogels. **(E)** Load and displacement curves of different hydrogels in adhesion shear test. **(F)** Tensile strength of hydrogels. **(G)** Compression strength of different hydrogels. **(H)** Adhesion strength of different hydrogels.

The light transmittance of the hydrogel is above 90% within the visible wavelength ranging from 400 nm to 800 nm, demonstrating excellent transparency (Figure 1A; Supplementary Figure S1), which could facilitate wound monitoring during therapy. Sufficient mechanical strength facilitates well fitness between hydrogel and wound, which is the perquisite for ideal wound dressing. Here, the mechanical strengths of the series of hydrogels xNMA-yTHMA were measured. This series of hydrogels exhibit excellent adhesion, flexibility and tensile properties (Figure 1B). The elongation at break of this series of hydrogels is more than 1,000% (Figure 1C; Supplementary Tabel S2). When the total monomer content was 35% and 40%, the hydrogels xNMA-yTHMA showed excellent tensile strength above 200 kPa (Figure 1F; Supplementary Tabel S2). It is worth noting that the addition of THMA can enhance the elongation at break and tensile strength of hydrogels when the total monomer concentration is unchanged, which may be attributed to the tri-hydroxyl structure of THMA. In addition, this series of hydrogels can withstand a large compression deformation of 70% without breaking, and the compression strength is higher than 280 kPa (Figures 1D, G; Supplementary Tabel S2). The adhesion properties of the hydrogels were evaluated by the lap shear experiments. The hydrogel with higher hydroxyl group concentration showed better adhesion strength, which was greater than 10 kPa (Figures 1E, H; Supplementary Tabel S2). Besides, the swelling rate of the hydrogels in PBS are measured as 475%–600%, suggesting that it could absorb a large amount of tissue osmotic fluid at the wound (Supplementary Figure S3). The hydrogels possess a loose and porous construction, allowing them to absorb plenty of water (Supplementary Figure S3). When the water is adsorbed into the pores, they form hydrogen bonds with the amide bonds and hydroxyl groups on the hydrogel chains, causing an increase in distance between the hydrogel chains and resulting in material expansion. In particular, hydrogel 35NMA-5THMA shows the best mechanical properties. The elongation at break is up to 1737%, and the tensile strength and compressive strength are 259 kPa and 453 kPa, respectively (Figures 1C–H; Supplementary Figure S2; Supplementary Table S2). The excellent mechanical properties of hydrogels xNMA-yTHMA are attributed to the formation of multiple hydrogen bonds between amide groups and hydroxyl groups.

### 2.2 Antibacterial properties of hydrogels *in vitro*

**FIGURE 2:**
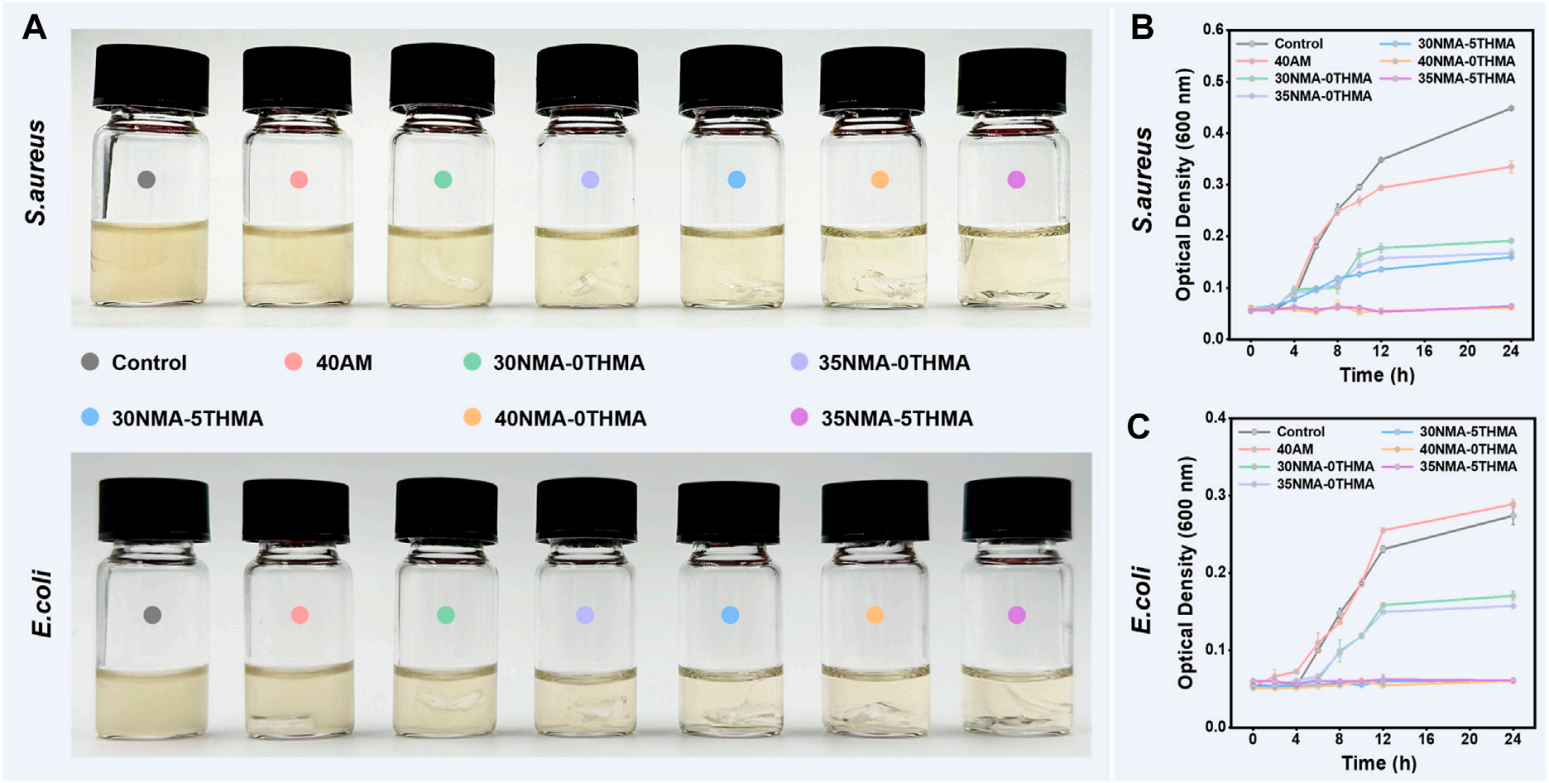
Evaluations of antibacterial properties of the hydrogels. **(A)** The photos of the culture medium containing *S. aureus* and *E. coli* after culturing with different hydrogel for 24 h. The growth curves of *S. aureus* **(B)** and *E. coli* **(C)** treated without (control group) or with different hydrogels in 24 h. The error bar is the standard deviation (*n* = 3).

Then, we explored the antibacterial effects of the above prepared hydrogels by the contact sterilization test against two common pathogenic microorganisms (*S. aureus* and *Escherichia coli*) in skin wounds. To verify the hypothesis that -OH contribute the bacterial killing efficacy, the 40a.m. hydrogel was used as a control, which is similar to xNMA-yTHMA does not contain the -OH. Firstly, the optical density data of culture media containing *S. aureus* and *E. coli*. were directly observed after co-culturing with different different formulation hydrogels for 24 h, with clearer media indicating higher inhibition efficiency (Figure 2A). The co-culture medium of the control and 40a.m. groups exhibited significant turbidity, indicating a substantial growth of *S. aureus*. In contrast, the co-culture medium of hydroxy-rich hydrogels 30NMA-0THMA, 35NMA-0THMA, 30NMA-5THMA, 40NMA-0THMA, and 35NMA-5THMA gradually became clear, suggesting the inhibition of bacterial proliferation. Among them, the co-culture medium of 40NMA-0THMA and 35NMA-5THMA was the clearest, suggesting the strongest inhibition of *S. aureus*. Similar results were observed in the treatment of *E. coli*, where 30NMA-5THMA, 40NMA-0THMA and 35NMA-5THMA exhibited the most potent inhibition effects. Subsequently, the growth curves of *S. aureus* and *E. coli* treated without or with different hydrogels in 24 h (Figures 2B, C). The bactericidal efficacy of 40a.m. is poor compared with other groups that contained NMA or THMA, suggesting -OH may account for the antibacterial property of the hydrogels. The inhibition efficacy of *S. aureus* was similar for 35NMA-0THMA and 30NMA-5THMA groups, which were slightly higher than 30NMA-0THMA, possibly due to more -OH. 40NMA-0THMA and 35NMA-5THMA almost completely suppressed *S. aureus* proliferation, as indicated by the marginal changes of OD600 from 0 to 24 h. For *E. coli*, the inhibition efficacy for 30NMA-0THMA and 35NMA-0THMA was feeblish. After increasing the -OH content of the hydrogels, the antibacterial effect of 30NMA-5THMA, 40NMA-0THMA and 35NMA-5THMA was significantly improved.

**FIGURE 3:**
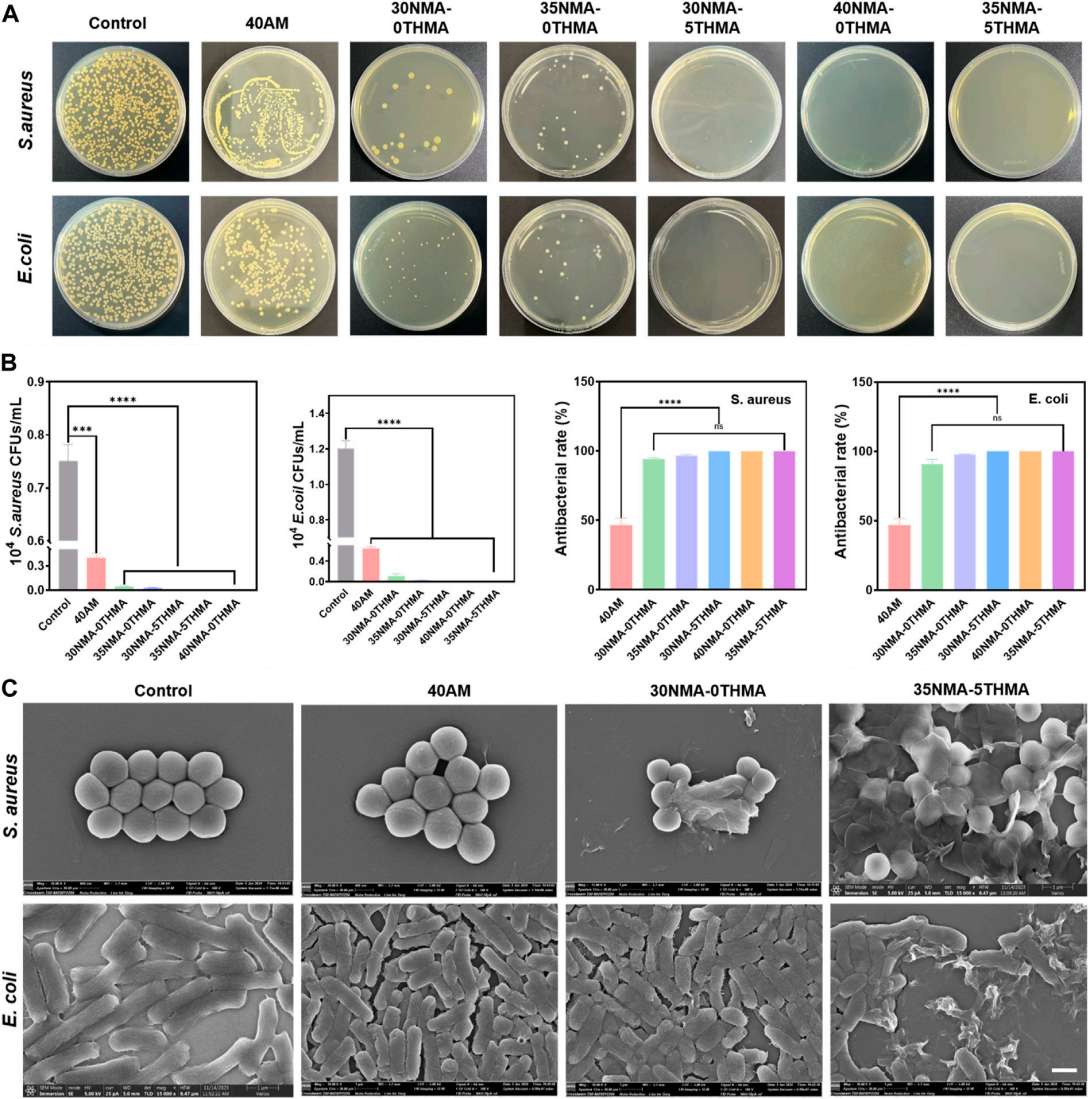
Evaluations of antibacterial properties of the hydrogels. **(A)** Photographs of the bacterial colonies of *E. coli* and *S. aureus* on agar plates after co-culturing without (control) and with different hydrogels for 24 h. **(B)** Quantitative results of the antibacterial properties of the hydrogels against *S. aureus* and *E. coli*. ****p < 0.0001. **(C)** SEM images of *E. coli* and *S. aureus* after co-culturing without (control) and with different hydrogels.

Furthermore, the standard plate-counting experiment was performed to evaluate the antibacterial properties of the hydrogels, as it could provide more distinctive comparison of the different hydrogels on decreasing bacteria colony. Compared to the dense colony distribution of the control groups, the number of colony-forming unit (CFU) of *S. aureus* and *E. coli* reduced to a certain extent after co-culturing with the hydrogel 40a.m. (Figure 3A). By statistical analysis, the bacteriostasis rate of 40a.m. is below 50%, indicating feeble antibacterial property of the hydrogel 40a.m. without -OH (Figure 3B). The drastic reduction of colony numbers occurred with the bacteriostasis rate above 90%, when hydroxy-rich xNMA-yTHMA were employed to co-culture with *S. aureus* and *E. coli*. Particularly, almost all *S. aureus* and *E. coli* lost viability and no colonies were formed after co-culturing bacteria with the hydrogel 30NMA-5THMA, 40NMA-0THMA and 35NMA-5THMA, demonstrating outstanding bacteriostatic performance (Figures 3A, B).

Furthermore, SEM revealed the morphology changes of *S. aureus* and *E. coli* after incubating with the hydrogels. For *S. aureus*, the morphology showed no significant changes compared with the untreated ones, and generally remained the normal spherical shape after treating with 40a.m. However, after treating with polyhydroxyl hydrogels (xNMA-yTHMA), the membrane structure of *S. aureus* gradually collapsed or even ruptured with the increase of hydroxyl content. The most severe rupture of the morphology was observed in the 40NMA-0THMA and 35NMA-5THMA groups, with most *S. aureus* collapsed into pieces. The structural deformation of *E. coli* also occurred when -OH was introduced in the hydrogels. The structures generally remained normal rod-shape treated with hydrogel 40a.m. and 30NMA-0THMA. Increase of -OH content led to bacterial deformation to a certain extent (35NMA-0THMA and 30NMA-5THMA). The most aggravated structural disruption was observed in 40NMA-0THMA and 35NMA-5THMA with highest -OH content. In these groups, most *E. coli* lost their rod-like shape, shrinking and collapsing into irregular fragments (Figure 3C; Supplementary Figure S4). The SEM results suggest that the polyhydroxyl hydrogels may inhibit bacteria by contacting and draining the intracellular substances, leading to the destruction of homeostasis.

Collectively, the above data strongly support that the -OH is responsible for the observed antibacterial effects in a concentration-dependent manner. The underlying mechanism of polyhydroxyl hydrogels for the sterilization may be that the hydroxyl groups on the hydrogel can interact with the bacterial membrane, resulting in the destruction of the membrane structure, thus effectively killing bacteria. Importantly, the excellent antibacterial effects were achieved exclusively by the intrinsic property of the hydrogel without loading of external agents. Compared with conventional hydrogels loaded with exogenous antimicrobial agents (such as antibiotics, metal ions, cationic antimicrobials, natural antimicrobials, etc.), these simple polyhydroxyl hydrogels with inherent antimicrobial properties may provide more favorable bio-compatibility.

### 2.3 Biocompatibility of the hydrogels

**FIGURE 4:**
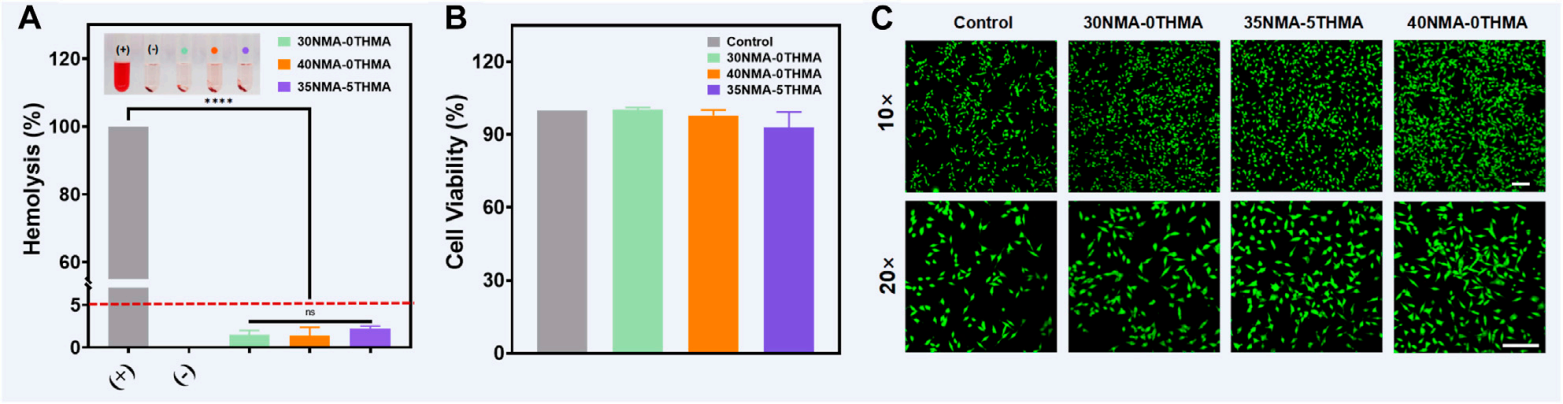
Biocompatibility characterization of hydrogels. **(A)** Hemocompatibility of hydrogels with different monomer concentrations. “+” and “−” represent positive control and negative control, respectively. The error bar is the standard deviation (*n* = 3). ****p < 0.0001. **(B)** CCK-8 assay of L929 cells for cell viability testing of the hydrogels after culturing 24 h. **(C)** Live/dead staining of L929 cells after 24 h of incubation with hydrogel. Scale bar: 100 μm.

As the prerequisite for biomedical applications, the safety of the antibacterial hydrogels 30NMA-0THMA, 40NMA-0THMA and 35NMA-5THMA was evaluated in respect to the hemolysis and cell viability. Encouragingly, the tested formulations with potent bacteria-killing efficacy showed negligible influence on the integrity of red blood cells. The hemolysis rates of 30NMA-0THMA, 40NMA-0THMA and 35NMA-5THMA were below 5%, the threshold for safety use (Figure 4A). Moreover, the above hydrogels could accelerate blood clotting, as indicated by the lower dynamic coagulation index (BCI) compared with the gauze and negative controls (Supplementary Figure S5). In addition, The *in vivo* hemostatic capacity of the hydrogels was examined in a mouse model of liver bleeding. Application of the hydrogel 35NMA-5THMA to the bleeding site of the liver significantly reduced blood loss (Supplementary Figure S6). The excellent antibleeding effect of the hydrogel could be attributed to the physical blocking of bleeding defects by the 3D hydrogel network and the adhesion of the hydrogel to the tissue surface. At the same time, the positively charged amino groups on the hydrogel interact with the negatively charged platelets by electrostatic force, thus activating the blood clotting process. The Cell Counting Kit 8 (CCK-8) was then used to assess the impact of the multifunctional hydrogel on the activity of mouse fibroblasts (L929). As shown in Figure 4B, after 24 h of co-culture with the hydrogel, there was no significant difference in cell survival data compared to the blank control. Subsequently, the survival of L929 cells were directly observed by the living/dead staining after 24 h of co-culture with the hydrogel. The living cells with intact membranes were stained with calcein-AM (green signal), and the dead cells were labeled with PI (red signal). The cells in all groups demonstrated bright green fluorescence with no noticeable red signal, and the observation at higher magnification found no obvious morphological changes (Figure 4C), confirming the excellent biocompatibility of the multifunctional hydrogel for wound dressings. Overall, the hemostasis and biocompatibility properties synergistically to support the translational potential of composite hydrogels in wound healing.

### 2.4 *In vivo* wound healing

**FIGURE 5:**
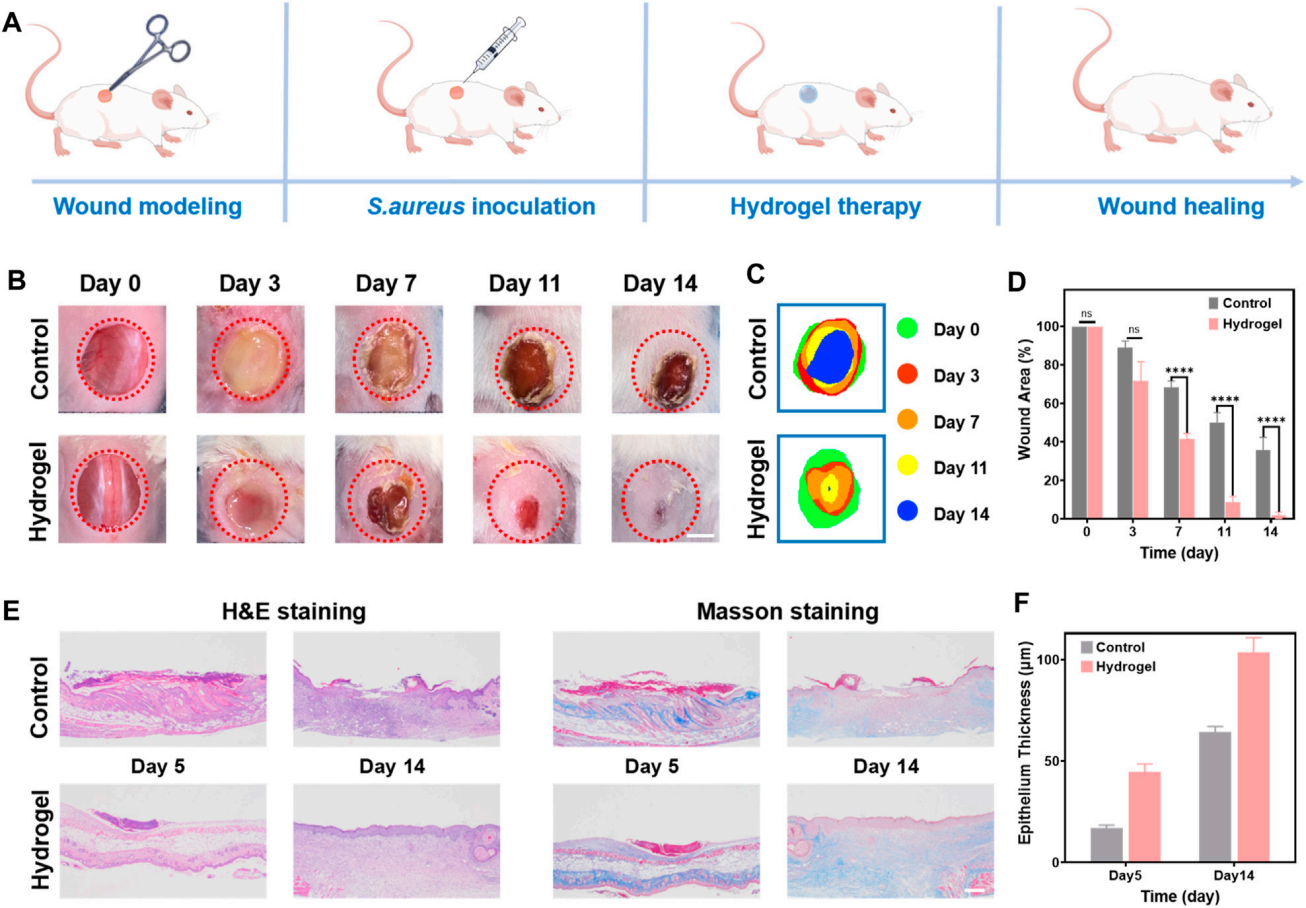
*In vivo* wound healing performance of the hydrogel. **(A)** Experimental procedures using hydrogels for wound treatment. **(B)** Photographs of skin wound treated with PBS (control) and hydrogel dressing from day 0 to day 14, respectively. Scale bar: 5 mm. **(C)** Schematic diagram of the wound area of control and hydrogel for 14 days. **(D)** Evolution of wound area on day 0, 3, 7, 11, and 14. The error bar is the standard deviation (*n* = 3). ****p < 0.0001. **(E)** H&E and Masson’s trichrome staining of wound tissues on day 5, and 14 for different treatments. Scale bar: 100 μm. **(F)** The epithelium thickness of wound tissues on day 5, and 14 for different treatments. The error bar is the standard deviation (*n* = 3).

A full-thickness wound infection mouse model using *S. aureus* was constructed to evaluate the healing efficacy of a composite hydrogel as a wound dressing (Figure 5A). The hydrogel 35NMA-5THMA exhibiting satisfying biocompatibility, mechanical properties, and antimicrobial efficacy, was selected to treat the infected wounds. The wound closure of the mouse of the control group was significantly slower than that in the hydrogel-treated group. At day 14, while large wound area still existed in the control group, which was even deepened as the deterioration of bacterial infection (Figure 5B). In sharp contrast, almost complete healing of the wound was achieved after hydrogel therapy. Measurement of the wound area throughout the entire healing process showed that the wound closure rate in the hydrogel group reached 100%, in contrast to the 62% of the control group (Figure 5C; Supplementary Figure S7). The calculation of wound areas was consistent with the observation (Figure 5D). The wound healing rates of hydrogel group and control group are calculated as 3.59 cm^2^/d and 2.16 cm^2^/d, respectively, that is 1.67-fold compared to control group, which is superior to the reported hydrogel dressings (Supplementary Table S1). The accelerated healing is most possibly due to the potent bacterial inhibition and hemostatic capacities of the hydrogels. Taken together, the results of these *in vivo* experiments confirm the excellent wound healing efficacy *in vivo*. Further, hematoxylin-eosin (H&E) staining and Masson’s trichrome staining were used for histological analysis of the wounds on day 5 and day 14 (Figure 5E). H&E staining images in the treatment group showed dense connective tissue, reduced inflammation, and thickened epithelium compared to the control group. On day 14, the wound’*s epidermis* was almost completely healed, with a thickness of 106.4 μm (Figure 5F). Masson’s staining shows that the amount of regenerated collagen in the wound gradually increases with the passage of time during the healing process (blue). Compared to the control group, the collagen deposition in the hydrogel dressing group was the highest and densest. This finding further supports the idea that the composite hydrogel can effectively enhance wound healing when used as a wound dressing. In addition, histological analysis of mouse organs (liver, spleen, kidney, heart, and lungs) showed no noticeable organ damage after 14 days of treatment in mice (Supplementary Figure S8).

## 3 Conclusion

In a word, hydrogels were prepared by free radical polymerization of hydroxyl-functionalized acrylamide monomers, NMA and THMA. According to the varying concentrations of NMA and THMA, the hydrogels were prepared and named as xNMA-yTHMA (30NMA-0THMA, 35NMA-0THMA, 30NMA-5THMA, 40NMA-0THMA, and 35NMA-5THMA). These physically cross-linked hydrogels exhibited excellent tensile properties, adhesion, and swelling, which may be attributed to the ability of hydrogen bonds to break and recombine. The incorporation of THMA enhanced the mechanical properties of the hydrogels. Particularly, 35NMA-5THMA possess high tensile strength of 259 kPa, large tensile strain of 1737%, and ideal compressive property. *in vitro* antibacterial experiments demonstrated that the antibacterial property of hydrogels xNMA-yTHMA gradually enhanced with the increase of NMA and THMA monomer content, that is, the increase of hydroxyl content. Hydrogels 40NMA-0THMA and 35NMA-5THMA showed remarkable and long-lasting antibacterial capabilities to the common pathogenic microorganisms *S. aureus* and *E. coli*. In addition, the hydrogels displayed good blood and cell compatibility according to blood compatibility tests, live/dead cell staining, and CCK-8 assays, as well as sufficient hemostatic ability for treating skin wounds. Moreover, after treating mice wounds with hydrogel 35NMA-5THMA, the wound healing rate was significantly faster (1.67-fold) compared to the BPS group, and no inflammatory reaction was observed. Besides, collagen deposition increased, and almost no scar formation or apparent organ damage was observed after 14 days of treatment. This suggests that the hydrogel dressing based on the hydroxy-rich hydrogel 35NMA-5THMA can effectively kill bacteria, reduce inflammation, and promote healthy epidermal regeneration. This hydroxy-rich inherent antibacterial material holds great promise as a wound repair material.

## 4 Experimental

### 4.1 Materials and instrumentation

N-methylacrylamide (NMA) (purity 98%) was obtained from Shanghai Energy Chemical Company, while N-tri (hydroxymethyl) acrylamide (THMA) (purity 98%) and 2,2′-azo [2-(2-imidazole-2-yl)propane]dihydrochloride (AIBI) (purity 98%) were acquired from Shanghai Bide and Shanghai Shawn respectively. *S. aureus* (*S. aureus*, BNCC 186335) and *E. coli* (*E. coli*, BNCC 13264), collected from BeNa Culture Collection, were cultured in nutrient broth (NB) and nutrient agar (NA) medium purchased from Hope Biotech in Qingdao, China. Mouse fibroblasts (L929), purchased from BeNa Culture Collection, were cultured in Dulbecco’s modified medium supplemented with fetal bovine serum obtained from GIBCO fetal bovine serum in Australia and trypsin. Live/dead staining reagents calcein-AM (AM) and propyl iodide (PI) were purchased from Bidder and their working concentrations were 2 μM and 10 μM, respectively. Unless otherwise stated, all reagents were used as received without further purification. All animal experiments strictly followed the guidelines of the National Regulations on the Care and Use of Laboratory Animals in China and were approved by the Animal Ethics Committee of Hainan University. BALB/c female mice aged 6–8 weeks weighing between 18–20 g each were purchased from SJA Laboratory Animal Co., Ltd., Hunan; they were housed in a temperature-controlled room maintained at 22°C ± 2°C with daily monitoring of their water intake and diet. The UV-visible transmission spectra were measured by a UV-visible spectrophotometer (Shimadzu, UV2600i, Japan). The mechanical properties of the hydrogels were evaluated using a universal testing machine (Shimadzu, AGS-X, Japan). The appearance of bacteria was observed by emission scanning electron microscopy (Thermo Scientific, Verios G4 UC, US). The optical density (OD600) values of the above bacterial solutions were measured with a microplate reader (Molecular Devices, SpectraMax iD3, Shanghai). Live/dead staining assay was observed under a laser confocal microscope (FV1200, Olympus, Japan).

### 4.2 Synthesis of hydrogels

We developed a composite hydrogel dressing by combining N-methylacrylamide (NMA) and N-tri (hydroxymethyl) acrylamide (THMA). In short, we dissolved N-methylacrylamide and N-tri (hydroxymethyl) acrylamide in phosphate buffered saline (PBS) to create a composite solution. Next, we dissolved the composite solution in the ultrasonic cleaning machine for 30 min and thoroughly mixed it. We then add the initiator AIBI (0.25 wt% of NMA and THMA) and mix thoroughly. The resulting solution is transferred to a special mold and placed in an oven at 40°C to form a gel. Hydrogels are named 30NMA-0THMA, 35NMA-0THMA, 30NMA-5THMA, 35NMA-5THMA, and 40NMA-0THMA, depending on their monomer concentration.

## Data Availability

The raw data supporting the conclusion of this article will be made available by the authors, without undue reservation.
