# Supplementary material for: Polyhydroxy structure orchestrates the intrinsic antibacterial property of acrylamide hydrogel as a versatile wound-healing dressing
Source: Front Bioeng Biotechnol. 2024 Apr 24;12:1396892. doi: 10.3389/fbioe.2024.1396892 (PMC11076666; doi:10.3389/fbioe.2024.1396892)
Supplement: Supplementary file 1 [file DataSheet1.pdf]

Supplementary Material

Polyhydroxy Structure Orchestrates the Intrinsic Antibacterial Property of Acrylamide Hydrogel as a Versatile Wound-healing Dressing

Lu Zhang[1,#], Yu-Jiao Tang[1,#], Wen-Qing Zhang[1,#], Jian Wang[1], Yu-Jian Cai[1], Tian-Yi Qin[1], Deteng Zhang[2,*], Zhao-Hui Wang[1,*], Ya-Long Wang[1,*]

[1]State Key Laboratory of Digital Medical Engineering, Key Laboratory of Biomedical Engineering of Hainan Province, School of Biomedical Engineering, Hainan University, Sanya, Hainan, 572025, China

[2]Institute of Neuroregeneration and Neurorehabilitation, Qingdao University, Qingdao 266071, China

#These authors contribute equally to this work.

***Correspondence:**

zhangbio137@qdu.edu.cn; wangzhaohui@hainanu.edu.cn; ylwang@hainanu.edu.cn

1 Experimental

1.1 Characterization of the hydrogel

The UV-visible transmission spectra of hydrogels with a 1 mm thickness were measured between 400-800 nm using a UV-visible spectrophotometer (Shimadzu, UV2600i, Japan). The swelling rate of the hydrogels was assessed by submerging them in a solution of phosphate buffer saline (PBS) until swelling equilibrium was reached at room temperature. Subsequently, we calculated the swelling ratio according to the formula:

$$\text{Swelling ratio} = (W_t - W_0)/W_0 \times 100\%,$$

where W_0 and W_t denote the weight of the initial and swollen hydrogel, respectively.

The hydrogel was rapidly frozen in liquid nitrogen, followed by freeze-drying in a freeze dryer. The dried hydrogel was then taken out and fractured to obtain cross-sections. The fractured hydrogel was observed using a field emission scanning electron microscope (Thermo Scientific, Verios G4 UC) after sputter-coating with gold.

The mechanical properties of the hydrogels were evaluated using a universal testing machine (Shimadzu, AGS-X, Japan). The hydrogel samples were shaped into a dumbbell for tensile testing with a uniform length of 16 mm, a width of 4 mm, and a thickness of 2 mm. Technical terms are explained upon initial use for greater clarity. Applying a fixed tensile rate of 50 mm/min, a 50 N transducer was utilized. The hydrogel's tensile strength was determined as the maximum stress at which the sample was pulled off, and the corresponding strain represents the elongation at break. For compressive testing, cylinders were formed from hydrogels with a diameter of 10 mm and a height of 5 mm. The compression speed was set to 2 mm/min and the maximum pressure limit was restricted to 45 N to avert any repercussions that could potentially occur from compression disc application on the sensor and to safeguard the instrument. The elastic deformation of the hydrogel when 45 N of force was applied was considered as the compression strain.

The adhesion property of the hydrogels was evaluated by lap shear test. The cuboid hydrogel sample (20×20×1 mm) is placed between two pieces of glass. Then use 100 grams of weight to press the overlapping position for 5 min. All experiments were carried out at a steady drawing speed of 5 mm/min. The adhesion stress is calculated by dividing the maximum load by the adhesion area.

1.2 Antimicrobial properties of hydrogels tested by plate counting method

The bacteriostatic effect of composite hydrogel against *S. aureus* and *E. coli* was evaluated by counting viable bacteria colony-forming units. Specifically, *S. aureus* and *E. coli* were placed in NB liquid medium respectively and cultured in oscillating incubators at 37 °C and 120 rpm/min until the logarithmic growth stage for reserve use. The bacterial solution was diluted to 10^4 CFU/ml respectively for use, and the composite hydrogel was irradiated by ultraviolet light for use. Weigh 0.1 g of sterilized hydrogel and place it in a sterile centrifuge tube, add 100 μL of diluted bacterial solution and place it in an oscillating incubator for 1h, then add a certain amount of PBS, and aftershock centrifugation (no treatment was done in the control group), apply 100 μL bacterial solution evenly on the nutrient agar medium plate and incubate for 24 h. The colonies were photographed and counted for antibacterial performance analysis.

The appearance of bacteria was observed by emission scanning electron microscopy (Thermo Scientific, Verios G4 UC, US). Specifically, *S. aureus* and *E. coli* were placed in NB liquid medium respectively. It was cultured in an oscillating incubator at 37 ℃ and 120 rpm/min until logarithmic growth. The bacterial solution was diluted to 10^7 CFU/ml respectively for use, and the composite hydrogel was irradiated by ultraviolet light for use. 0.1 g of sterilized hydrogel was weighed and placed in a sterile centrifuge tube, then 100ul of diluted bacterial solution was added into an oscillating incubator for 1h, and then a certain amount of PBS was added (no treatment was done in the control group). Aftershock centrifugation, the supernatant was poured away, a certain amount of 2.5% glutaraldehyde was added for morphology fixation, and the gel was placed at 4 ℃ for 4 h. The supernatant was then dehydrated with 20%, 50%, 70%, and 100% ethanol in turn. The treated bacterial suspension droplets were added to the silicon wafers, and the samples were sprayed with gold after air drying.

1.3 Growth curves of bacteria and fungi co-incubated with hydrogels

To study the growth curves of *S. aureus* and *E. coli* under the conditions of co-culture with hydrogel, *S. aureu*s and *E. coli* were placed in NB liquid culture medium in a shaking incubator at 37 °C and 120 rpm/min. Culture until the logarithmic growth phase before use. Dilute the bacterial solution to 10^7 CFU/mL for later use. The composite hydrogel needs to be irradiated by ultraviolet rays and put into a sterile glass bottle for later use. *S. aureus* and *E. coli* suspensions (3 mL) were added to small glass bottles containing hydrogel. In this experiment, the bacterial solution without any treatment was used as the control group. Each sample was repeated three times. Bottles of bacteria were incubated at 37 °C and 120 rpm for 0–24 h. During the incubation process, the optical density (OD600) values of the above bacterial solutions were measured with a microplate reader (Molecular Devices, SpectraMax iD3, Shanghai) at different times, and the turbidity changes of the bacterial solutions at different times were observed.

1.4 Hemolysis rate test for hydrogels

Fresh heparinized whole blood was placed in a centrifuge tube with a specific volume of saline, shaken gently, and centrifuged. The supernatant was aspirated carefully using a pipette gun, and the washing process was repeated 2-3 times. The bottom erythrocytes obtained after centrifugation were resuspended in a 10% erythrocyte solution using saline. Technical term abbreviations such as 'erythrocyte' were explained upon first use. Biased or subjective evaluations were avoided, and a clear, concise, and logical structure was maintained while adhering to conventional academic formatting and style guidelines. A specific number of pure erythrocytes were added to a specific amount of distilled water to be a positive control. Additionally, 10% erythrocyte solution was added to a specific amount of saline to serve as a negative control. The fresh hydrogel was soaked in PBS for 24 h and left to stand. Subsequently, the experimental group added the same amount of 10% erythrocyte solution as the negative control and followed with an equal amount of hydrogel. Prepare three centrifuge tubes for each group and perform three controls. Incubate in a constant temperature box set to 37 ℃ for 1-1.5 hours. After that, remove the centrifuge tubes and subject each tube to centrifugation at 2500 rpm for 5 minutes. Take the 96-well plate and inject 100 μL of the supernatant into each healthy plate. Inject one well for each centrifuge tube. The positive group, the negative group, and the sample group supernatants should be injected into the healthy plate. Place the 96-well plate in the enzyme labeling machine (SpectraMax iD3, Molecular, Shanghai), and measure the absorbance of each well at 540 nm; the formula calculated the hemolysis ratio:

$$HR= (OD_{hydrogel} - OD_{negative})/ (OD_{positive} - OD_{negative}) \times 100\%,$$

where $OD_{hydrogel}$, $OD_{negative}$, and $OD_{positive}$ denote the measured absorbance of the hydrogel sample, negative control, and positive control, respectively.

1.5 *In vitro* coagulation assay with hydrogels

The hydrogels underwent testing for BCI index, in which lower values indicate superior coagulation properties. Technical abbreviations will be explained upon first usage. From healthy mice, whole blood was extracted from the eyeballs and treated with anticoagulant. The hydrogels were prepared as thin 8mm x 1mm slices, with gauze cut to the same size for comparison as a positive control. The hydrogel or gauze was warmed to 37 °C before incubating with 10 μL of recalcified whole blood (containing 10 μM $CaCl_2$) to initiate coagulation. Following incubation at 37 °C for 5, 10, 20, and 30 min, 1.0 mL of deionized water was gently introduced to the mixture to disrupt free erythrocytes. After centrifugation at 2500 rpm for 5 min, hemoglobin absorbance in the supernatant was measured at 540 nm using a microplate apparatus. BCI was calculated by the following equation:

$$BCI\ (\%) = A_{sample} / A_{control} \times 100\%,$$

where A_{sample} and $A_{control}$ represent the absorbance of the sample and control (10.0 μL recalcified whole blood dissolved in 1.0 mL deionized water), respectively.

The hydrogel's hemostatic properties were evaluated using a mouse liver hemorrhage model. Briefly, mice were anesthetized, the abdomen was cut open to expose the liver, and tissue fluid around the lesion was carefully removed with cotton swabs. The liver was placed on a pre-weighed filter paper (W_0), and the hemorrhage was cut with a scalpel. The complex hydrogel and gauze were placed on the bleeding site respectively. The untreated wound was used as the control group. Filter paper weight (W_t) was measured 60 s after bleeding. The amount of blood loss is calculated by increasing the weight of the filter paper.

1.6 Biocompatibility evaluation of the hydrogels

The biocompatibility of the multifunctional hydrogels was assessed by measuring the survival rate of L929 mouse fibroblasts through the CCK-8 cell viability assay. The hydrogel was immersed in PBS over three days to eliminate impurities. The solution was altered daily, and the hydrogel was then submerged in a 75% ethanol solution to extract misplaced bacteria. The hydrogel was soaked in PBS, replacing the alcohol, and ultimately exposed to UV irradiation for thirty minutes. A specific quantity of the hydrogel was placed in an incubator at 37 °C with DMEM medium for twenty-four hours of co-incubation, all to gain the hydrogel leachate. L929 cells were inoculated into 96-well plates at a density of 20,000 cells per well. Subsequently, 100 μL of DMEM medium was added to each well of the control group, whereas the experimental group was treated with different hydrogels and their respective leachates following incubation. After a total incubation period of 24 h, 10% CCK-8 reagent was added and incubated for another 2 hours. The optical density at 450 nm (OD450) was then measured using an enzyme counter, and the cells' survival rate was calculated.

Live/dead staining assay: L929 cells were cultured in confocal dishes at a density of 80,000 cells per dish and incubated with hydrogel leachate for 24 h. Following this, the calcian AM and PI staining kit (Macklin, China) was introduced to the dishes and incubated for 20 min. Subsequently, the staining solution was removed, and the cells were washed thrice with cold PBS before being stained and observed under a laser confocal microscope (FV1200, Olympus, Japan). Live/dead staining

reagents calcein-AM (AM) and propyl iodide (PI) were purchased from Bidder and their working concentrations were 2 μM and 10 μM, respectively. The excitation wavelength of calcian AM is 488 nm, and the collection of emission wavelength is 500-530 nm. The excitation wavelength of PI is 514 nm, and the collection of emission wavelength is 580-650 nm.

1.7 In vivo wound healing experiments

Mice received an intraperitoneal injection of an anesthetic comprising a 2.5% tribromoethanol solution (Sigma, USA) at a dose of 100-200 μL/10 g. Under aseptic conditions, a standardized full-thickness skin wound (8 mm diameter) was created on the back of each mouse by excising a portion of the skin using a biopsy punch after partial hair removal with depilatory cream. Next, a suspension of *S. aureus* with a concentration of 10^8 CFU/mL was administered to the wound. One wound was covered with hydrogel that had been prepared beforehand and secured using medical tape, while the other wound was used as a control and secured with medical tape alone. Photographs of all wounds were taken on days 0, 3, 7, 11, and 14 to document the condition of the wound, and the rate of wound healing was calculated by determining the ratio of the healed area to the original area after completion of treatment.

Samples of skin tissue were taken on days 5 and 14, respectively. Skin tissue samples were fixed in 4% paraformaldehyde for one hour before being embedded in paraffin and sectioned. Hematoxylin and eosin (H&E) as well as Masson's trichrome stain were used to stain all tissue sections. The sections were observed and photographed using light microscopy.

Mice were euthanized on day 14, and heart, liver, spleen, lung, and kidney samples were collected. The samples were then stained with Hematoxylin&Eosin (H&E) and photographed for analysis.

2 Supplementary Tables and Figures

2.1 Supplementary Tables

Table S1 Summary of antibacterial hydrogels reported recently

Hydrogel	Antibacterial agent	Antibacterial rate	Tensile strain (%)	Tensile strength (kPa)	Transparency	Wound healing rate of hydrogel group (cm^2/d)	Wound healing rate of control group (cm^2/d)	Ratio[a]	Ref
BCD/PDA/PAM	BCD	99%	1047	21-51	Tawny	3.34	2.84	1.17	[1]
CUR/TA	TA	99%	×	×	Tawny	3.2	2.87	1.11	[2]
PAA/GelMA/CuS@LAS	CuS@LAS NPs	>90%	×	×	Milk white	2.83	1.88	1.5	[3]
OSD/CMC/Fe/PA	Photothermal fungicide	100%	×	×	Black	3.40	2.87	1.18	[4]
QP/NMN/Mg2+	QCS/ Mg2+	>90%	×	×	Bronzing	3.51	2.15	1.63	[5]
PZBA−PVA	QAs	100%	×	×	Milk white	2.51	2.15	1.16	[6]
Jelleine-1	Natural antimicrobial peptide	100%	×	×	Transparent	3.58	3.36	1.06	[7]
SIS/ZIF	Zn2+	>90%	38	28000	White film	4.10	3.76	1.09	[8]
IPSPKa	Ga2+	99%	×	×	Spray	8.83	7.94	1.11	[9]
HACC-PAM	HACC	>80%	145	30	Transparent (95%)	×	×	×	[10]
GA-QCS/OHA	QCS	>90%	×	×	Transparent	3.65	3.07	1.19	[11]
This work	-OH	100%	1737	258.68	>90%	3.59	2.15	1.67	

Table S2 Mechanical properties of xNMA-yTHMA hydrogels

Sample	Elongation at break (%)	Tensile strength (kPa)	Compression strength at 70% strain (KPa)	Adhesion strength (kPa)
30NMA-0THMA	1286	123	284	4
35NMA-0THMA	1090	202	287	8
30NMA-5THMA	1252	227	302	7
40NMA-0THMA	1514	231	393	12
35NMA-5THMA	1737	259	453	17

2.2 Supplementary Figures

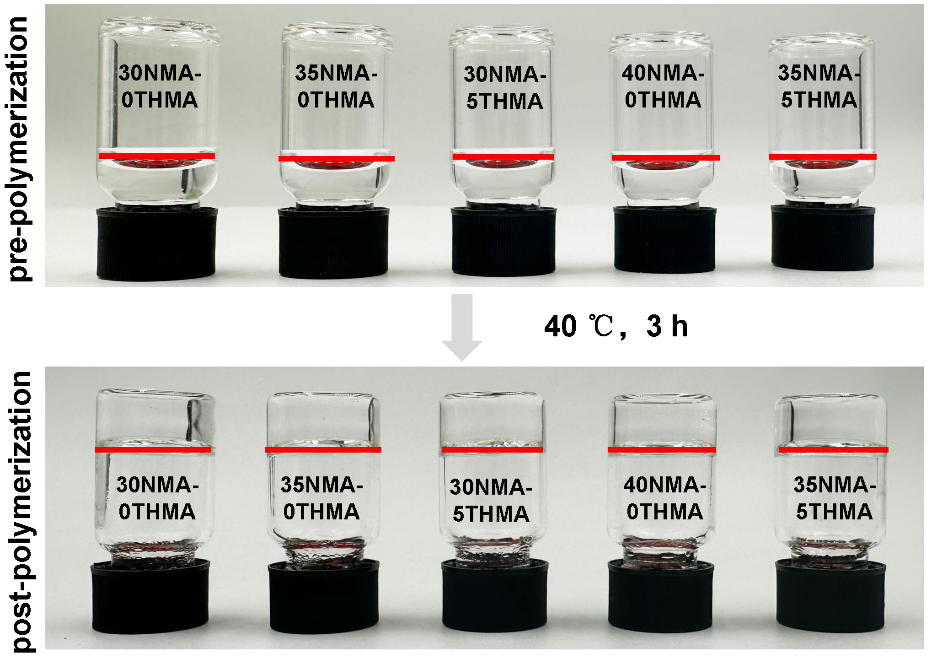

Fig. S1 Photographs of hydrogels before and after gelation.

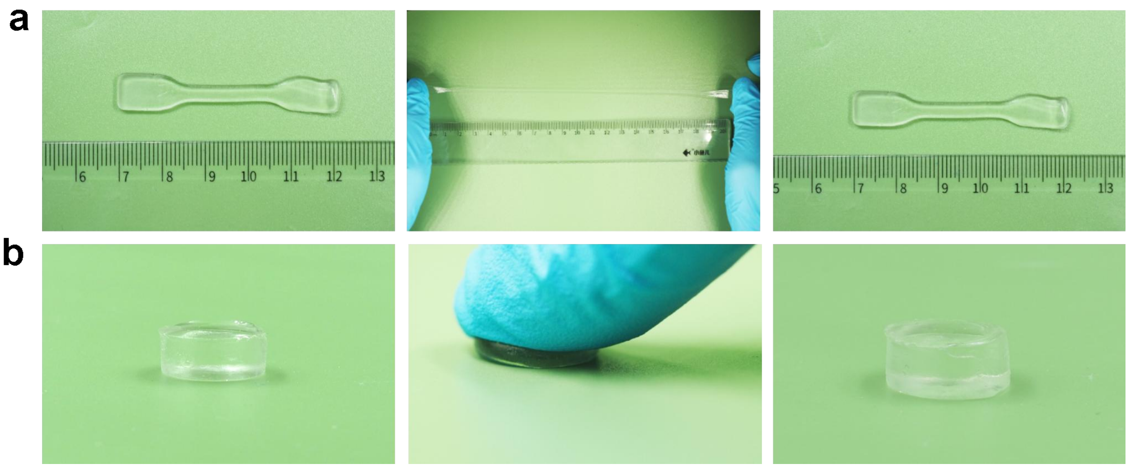

Fig. S2 (a) Photographs of the tensile properties of hydrogel 35NMA-5THMA. (b) Photographs of compression properties of hydrogel 35NMA-5THMA.

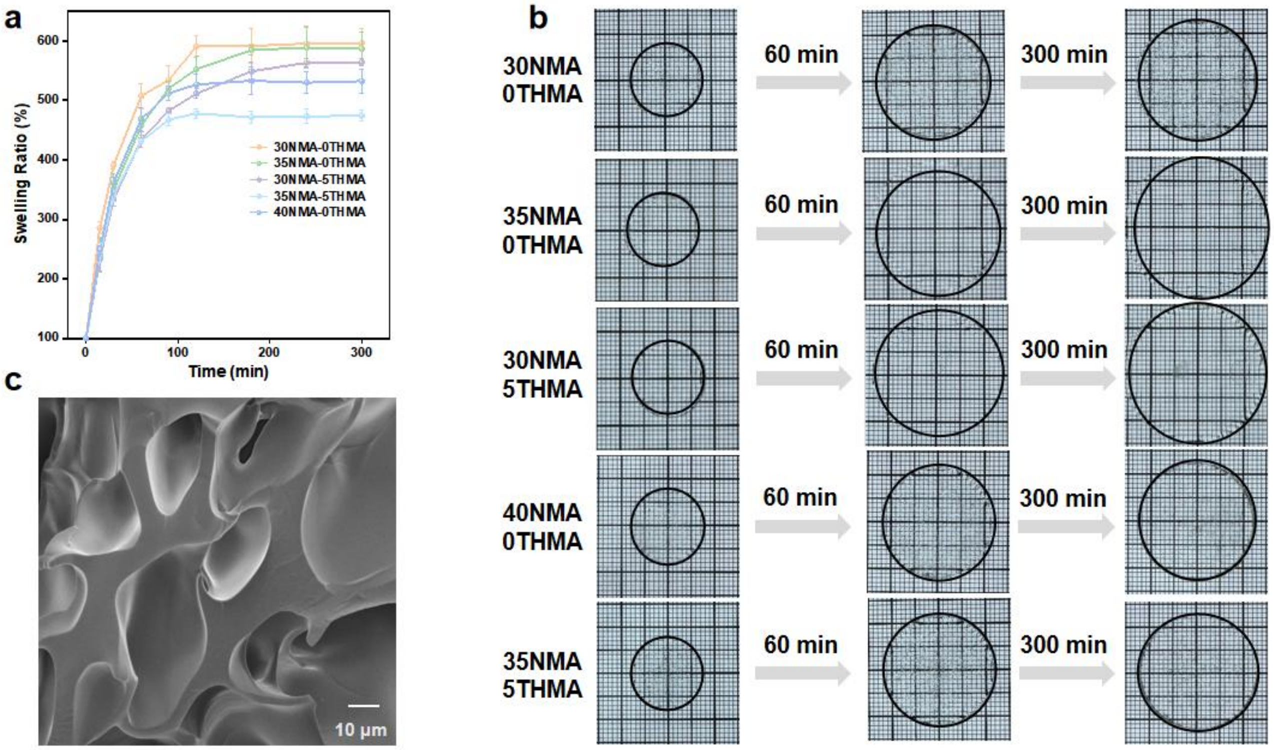

Fig. S3 (a) The swelling ratio of hydrogels at 37 °C in PBS (pH 7.4) for 300 minutes. (b) Photographs of swelling process for hydrogels at 37 °C in PBS (pH 7.4). (c) SEM images of hydrogels. Scale bar:30 μm.

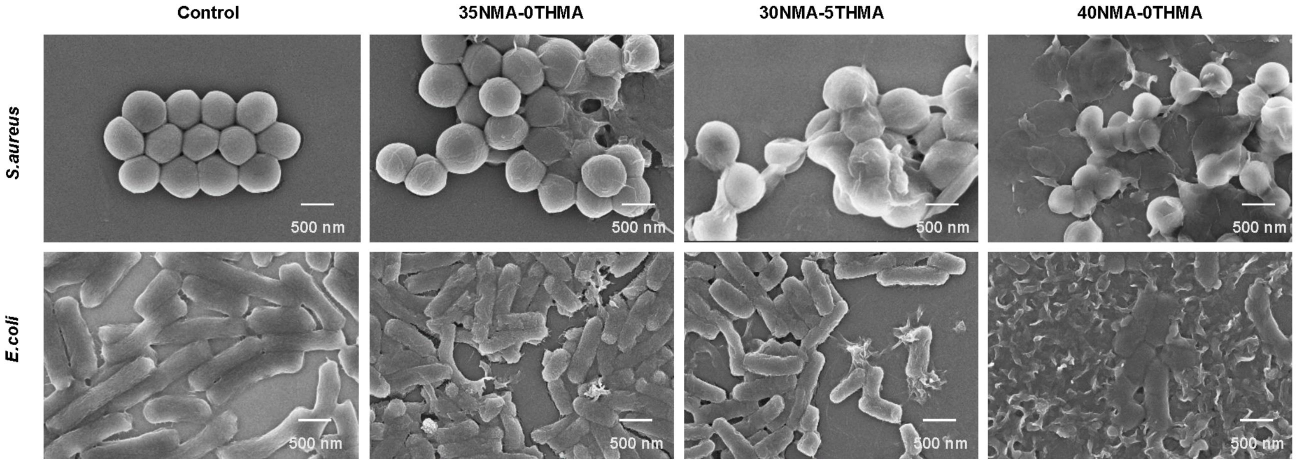

Fig. S4 SEM images of *E. coli* and *S. aureus* after co-culturing without (control) and with different hydrogels.

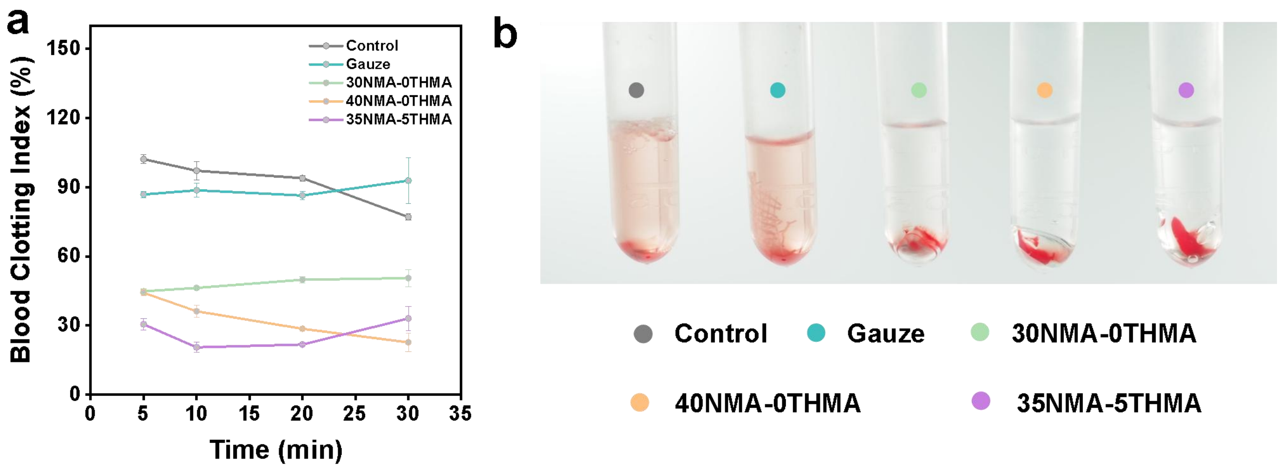

Fig. S5 (a) The dynamic blood-clotting index of different samples. (n=3) (b) Photographs of blood-clotting effect of different samples.

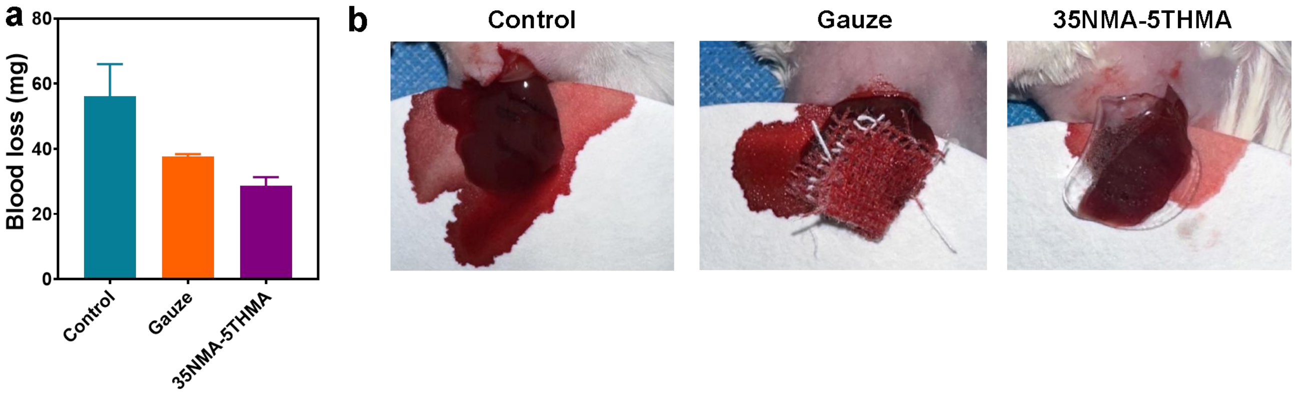

Fig. S6 (a) Amount of liver blood lost in the 60 s by different treatments. (n=3). (b) Representative Photographs of liver bleeding after different treatments within the 60 s.

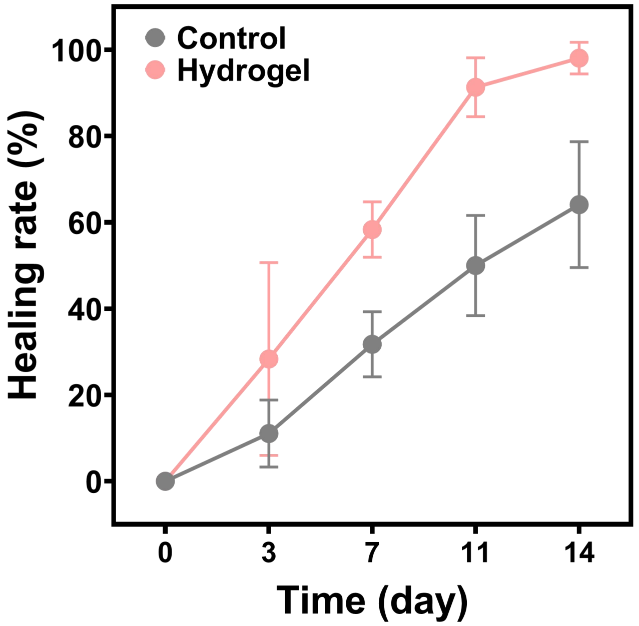

Fig. S7 The wound healing rates on day 0, 3, 7, 11 and 14 at different treatments. The error bar is the standard deviation (n = 3)

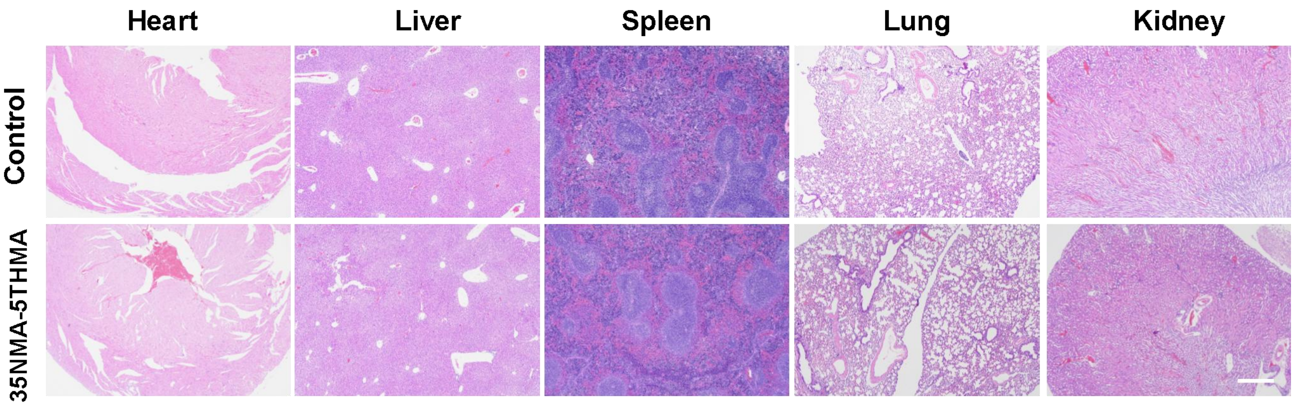

Fig. S8 H&E staining images of the main organs of mice with or without implantation of hydrogel 35NMA-5THMA on day 14. Scale bar: 200 μm.

3 References

[1] Z. Yang, R. Huang, B. Zheng, W. Guo, C. Li, W. He, Y. Wei, Y. Du, H. Wang, D. Wu and H. Wang, Highly Stretchable, Adhesive, Biocompatible, and Antibacterial Hydrogel Dressings for Wound Healing, Adv Sci (Weinh), 2021, 8, 2003627

[2] Z. Zhou, J. Xiao, S. Guan, Z. Geng, R. Zhao and B. Gao, A hydrogen-bonded antibacterial curdlan-tannic acid hydrogel with an antioxidant and hemostatic function for wound healing, *Carbohydr Polym*, 2022, **285**, 119235.

[3] D. He, C. Liao, P. Li, X. Liao and S. Zhang, Multifunctional photothermally responsive hydrogel as an effective whole-process management platform to accelerate chronic diabetic wound healing, *Acta Biomater*, 2024, **174**, 153-162.

[4] L. Qiao, Y. Liang, J. Chen, Y. Huang, S. A. Alsareii, A. M. Alamri, F. A. Harraz and B. Guo, Antibacterial conductive self-healing hydrogel wound dressing with dual dynamic bonds promotes infected wound healing, *Bioact Mater*, 2023, **30**, 129-141.

[5] J. L. Zhen Liang, Songmiao Liu, Yanan Gu, Zhiwei Cui, Yuhan Zhu, Zhou Yu, Xin Zhao, Baolin Guo, Baoqiang Song, Injectable, antibacterial, ROS scavenging and pro-angiogenic hydrogel adhesives promote chronic wound healing in diabetes via synergistic release of NMN and Mg^{2+}, *Chemical Engineering Journal*, 2023, **475**.

[6] Z. Zhang, Z. Ni, Y. Huang, H. Zhang, Z. Hu, D. Ye, Y. Shen, M. Jia, K. Shi, G. Zhu, J. He, L. Xu, F. Shi, H. Yu, L. Zhuang and H. Wang, Barnacle-Inspired Wet Tissue Adhesive Hydrogels with Inherent Antibacterial Properties for Infected Wound Treatment, *ACS Appl Mater Interfaces*, 2023, **15**, 37214-37231.

[7] R. C. Jingjing Zhou, Zhiyu Wu, Chunliang Zhang, Yuhang He, Hanru Zhang, and M. S. F. Kexin Liu, Zhaopeng Wang, Changyan Yang, Yifan Zhang, Wenjin Yan, Kairong Wang, An injectable, natural peptide hydrogel with potent antimicrobial activity and excellent wound healing-promoting effects, *Nano Today*, 2023, **49**.

[8] W. Y. Zelong Song, Xuesong Zhang, Yuan Dong, Zhen Zhang, Yiqian Huang, Wei Jing, Liya Sun, Yan Han, Fanqi Hu, Zuoying Yuan, Bo Zhao, Pengfei Wei, Xuelian Zhang, Controlled growth of metal-organic frameworks on small intestinal submucosa for wound repair through combined antibacterial and angiogenic effects, *Nano Today*, 2024, **54**.

[9] W. Guan, C. Gong, S. Wu, Z. Cui, Y. Zheng, Z. Li, S. Zhu and X. Liu, Instant Protection Spray for Anti-Infection and Accelerated Healing of Empyrosis, *Adv Mater*, 2024, **36**, e2306589.

[10] Lirong Wang, Mengyun Zhou, Tailin Xu and X. Zhang, Multifunctional hydrogel as wound dressing for intelligent wound monitoring, *Chemical Engineering Journal*, 2022, **433**, 134625.

[11] Q. Bai, Q. Gao, F. Hu, C. Zheng, W. Chen, N. Sun, J. Liu, Y. Zhang, X. Wu and T. Lu, Chitosan and hyaluronic-based hydrogels could promote the infected wound healing, *Int J Biol Macromol*, 2023, **232**, 123271.
